# Cosmetic Dermatology in the Digital Age

**DOI:** 10.3390/jcm13226953

**Published:** 2024-11-18

**Authors:** Aurore D. Zhang, Roxana Shirazi, Neelam A. Vashi

**Affiliations:** 1 Department of Dermatology, Boston University Chobanian and Avedisian School of Medicine, Boston, MA 02118, USA; auzhang@bu.edu; 2 Department of Dermatology, Virginia Commonwealth University Health System, Richmond, VA 23298, USA; roxana.shirazi1@vcuhealth.org

Like many other industries, healthcare is constantly evolving. Driven by rapid advances in technology and the ever-growing influence of the digital landscape, the art of caring for the physical body and the digital world are becoming increasingly intertwined. The field of cosmetic dermatology is no exception; in fact, it may exceedingly embody this entanglement. The marriage of innovation, social media, and telehealth consultations has changed how patients perceive and seek beauty and how they engage with dermatologists. As such, dermatologists must engage as active participants as cosmetic dermatology continues to grow in the digital age.

## 1. The Digitalization of Beauty: New Frontiers in Cosmetic Dermatology

Technologies such as artificial intelligence (AI), augmented reality (AR), and high-resolution imaging are powerful tools that are pushing the boundaries of what we can offer patients in cosmetic dermatology. Algorithms driven by AI can now analyze patients’ unique skin types, providing them with personalized skincare. Patients can utilize AR to simulate before and after images of cosmetic procedures, such as filler and laser treatments, in real-time. High-resolution imaging can detect early signs of aging, pigmentation, and changes in skin laxity, identifying them before they become more noticeable and while treatments are more effective and affordable. These tools empower patients by offering a tangible, visual sense of what they could potentially expect, helping them make informed decisions about their esthetic goals, bridging expectations, and improving both satisfaction and patient-physician relationships.

While the rise of these digital tools offers exciting possibilities, they are not without challenges. The flawless results and treatment plans AR and AI simulations portray can set unrealistic expectations that prove difficult to achieve in reality and in real time, making managing patient expectations trickier. While AI can generate recommendations based on an enormous amount of data in seconds, it cannot fully account for the complexity and unpredictability of human skin and response to treatment. As a result, dermatologists may find difficulty managing patients’ expectations that have been guided by these technologies to expect perfection or immediate results.

The digitization of beauty can and will continue to empower patients and enhance what we can accomplish with cosmetic dermatology, but it also highlights the importance of clear communication between patient and physician. Dermatologists must navigate the balance between the benefits of these tools and managing patient expectations by helping patients understand the value and limits of technology and the complexity of the human body.

## 2. The Social Media Effect: A Double-Edged Sword

With the rise of social media, the way that people interact with, share information, and consume content has fundamentally changed. Platforms like Instagram and TikTok have seen a huge growth in users fast-tracking the exchange of information like never before. Social media has developed an important presence in nearly every industry, and cosmetic dermatology is no exception.

Access to and information about cosmetic dermatology has increased through social media by offering before-and-after photos, real-time procedure videos, and a never-ending stream of discussion on the latest treatments and skincare recommendations. Social media users now regularly see cosmetic dermatology treatment results and procedures themselves, normalizing treatments like neurotoxin (i.e., Botox^®^), filler, and laser therapies. Information and personal experiences related to cosmetic dermatology treatments are at the fingertips of social media users. With a simple swipe or tap, patients can watch the timelapse of a filler appointment or learn about the newest laser treatments. Patients now often arrive at dermatology consultations already informed about a specific procedure, with the source easily accessible on their smartphone, highlighting social media as an educational tool for patients.

Social media, however, is not without its drawbacks. Social media influencers have become driving forces on social media platforms and often serve as a supposed trusted voice to thousands to millions of followers. They have also become powerful figures in shaping beauty standards and trends and can blur the lines of what is professional cosmetic dermatology advice. Influencers can promote skincare products or cosmetic procedures without background expertise, proliferating unqualified skin advice on social media that can potentially harm skin health. Social media users are also exposed to perfected, filtered images of influences and their peers, which fosters peer pressure to pursue unnecessary or premature cosmetic treatments, such as preventative neurotoxin or extensive skincare routines, in younger and younger individuals. Trends cycle quickly through social media, creating fleeting popularity for procedures, like masseter neurotoxin or lip flips, that may not be suitable for everyone. Despite these drawbacks, social media has also fostered positive communities for skin/body positivity. Users can find support and camaraderie in these virtual spaces, leading to people feeling more comfortable in their skin and embracing differences that may otherwise have been a source of insecurity.

## 3. Virtual Consultations and the Post-COVID Shift

The COVID-19 pandemic accelerated the development of telemedicine across the medical field, including cosmetic dermatology. Social distancing and lockdown measures pushed dermatologists to virtual consultations, revolutionizing how patients engage with their dermatologists and putting skin health, quite literally, at their fingertips. Virtual cosmetic consultations have made it more convenient for patients to discuss their cosmetic questions and goals and explore treatment options without having to account for time lost to travel or taking PTO. This has been especially beneficial for patients who reside in remote or underserved areas where specialists are few and far between.

While access to care has been expanded through telemedicine, dermatologists must be wary of the limitations of virtual consultations. Some conditions, consultations, and procedures require in-person to ensure safety and accuracy. Relying solely on virtual tools misses valuable information that can only be obtained by seeing the patient in person—such as the evaluation of skin texture and tone or the subtle ways a person’s face moves or crinkles. These details are critical in cosmetic dermatology in order to meet our patients’ goals and expectations, especially if pursuing a natural and seamless outcome. Therefore, it is the responsibility of dermatologists to balance the convenience of telehealth with the necessity of in-person exams to ensure the patient’s safety and cosmetic goals are best met.

## 4. Ethical and Regulatory Considerations

As the digital age ushers in new technologies and tools into the practice of cosmetic dermatology, it also introduces new ethical and regulatory challenges. Patients can now have online access to purchasing dermatological drugs and products, such as over-the-counter injectables, and can also consult providers overseas without fully understanding the associated risks. Products like neurotoxins and fillers are often sold online without proper regulation, putting patients at risk for unsafe use, improper application, or extremely harmful side effects. Accessing providers in foreign countries with varying medical standards through virtual consultations can leave patients vulnerable to variable, subpar care or a lack of consistent care and accountability. As such, cosmetic dermatologists have an important role to play in educating patients about the risks associated with self-administered treatments and unregulated services made accessible online. Individually, dermatologists can emphasize and recommend receiving procedures from licensed professionals who adhere to safety standards and ethical guidelines. On an institutional level, dermatologists should advocate for strong regulatory oversight of digital platforms to minimize misinformation and ensure patient safety. As both cosmetic dermatology information and procedures themselves become more accessible in the digital age, the need for greater awareness, patient education, and regulation grows more urgent.

## 5. Focus of Future Research in Cosmetic Dermatology

As cosmetic dermatology continues to evolve and change, future research will be vital in addressing both the opportunities and challenges the digital age brings. Artificial intelligence (AI) and machine learning present a promising area of research for personalized dermatological care. By analyzing diverse skin types and conditions through AI and machine learning algorithms, tailored treatment plans that consider genetics, environment, and lifestyle can become a reality. This technology is already being utilized commercially, with companies offering consumers personalized skincare recommendations based on data from extensive skincare databases. Other brands use augmented reality to allow customers to virtually “test out” products before purchasing. AI and machine learning are also being utilized in-office by dermatologists. The VISIA Skin Analysis System delivers a comprehensive skin analysis by analyzing wrinkles, UV damage, texture, pores, general spots, brown spots, red areas, and porphyrins. Initial research on this technology has found that 86% of patients reported that the VISIA analysis helped them understand their initial skin concern, 100% would recommend it to others, and 62% would prefer visiting a clinic that utilized the VISIA system [1]. Despite these amazing advances, AI is limited by the data it is trained on and thus reflects the lack of robust data on skin of color. As such, current AI programs do worse at analyzing skin of color [2]. More research in AI and machine learning in cosmetic dermatology is necessary to enhance the effectiveness and predictability of cosmetic procedures and promote safer and more inclusive practices across diverse populations.

In addition to technological advances, we must research the long-term effects of cosmetic procedures made popular by social media, such as fillers and lymphatic drainage, in order to better understand their safety and efficacy. Social media driven body dysmorphic disorder and social media dysmorphia are also important areas of research as they become increasingly prevalent, especially in our younger populations. Investigating the relationship between digital beauty standards and mental health and self-esteem will be vital in better supporting our patients and understanding the effects of social media on their psyche. Additionally, the myriad of ethical considerations that AI and social media influences bring forth must also be researched and described. What are the societal implications of digitized beauty standards? How can we protect patients from unethical and persuasive marketing tactics? The digital age and all its powerful tools must be the center of research so that we may not only better utilize it but also uphold ethical standards and foster a healthy relationship with beauty in the digital age.

## 6. Looking Ahead: The Future of Cosmetic Dermatology

The digital age offers an arsenal of exciting advancements and novel tools in the future of cosmetic dermatology. Diagnostic accuracy can be enhanced using AI by analyzing skin conditions and offering personalized treatment plans, while robotic assistance for procedures may streamline procedures, making them more precise and consistent. While these technologies have the potential to revolutionize cosmetic dermatology, it is essential to recognize and maintain the human element and artistry as the heart of dermatology. The nuanced, complex understanding dermatologists have of our patients’ needs, esthetic goals, and emotional well-being cannot be replicated by machines. As the field continues to intertwine with the digital age, it is crucial for dermatologists to uphold the warmth, empathy, and personalized care central to quality dermatological care alongside these advancements. The future will ultimately be shaped by balancing innovation with the art of dermatology so that patients receive effective and compassionate care.

## 7. Conclusions

Cosmetic dermatology in the digital age is rich with potential and intricate challenges. Dermatologists must learn to harness the power of technology while upholding the core values of safety, ethics, and patient satisfaction. Rapid advances in digital tools both enhance our ability to provide care and empower patients with information to make their own informed decisions about their skin health and cosmetic journeys. As we navigate this exciting transformation, we must proceed with responsibility and foresight. Technology should serve as a means of empowerment, not distortion, that celebrates individuality and authenticity. Ultimately, the future of cosmetic dermatology in the digital age lies in our hands—hands that will continue to skillfully deliver artistry and exceptional care for the well-being of our patients.
